# Ex-vivo drug screening of surgically resected glioma stem cells to replace murine avatars and provide personalise cancer therapy for glioblastoma patients

**DOI:** 10.12688/f1000research.135809.1

**Published:** 2023-08-08

**Authors:** Hannah Gagg, Sophie T. Williams, Samantha Conroy, Katie N. Myers, Connor McGarrity-Cottrell, Callum Jones, Thomas Helleday, Juha Rantala, Ola Rominiyi, Sarah J. Danson, Spencer J. Collis, Greg Wells

**Affiliations:** 1 Oncology & Metabolism, The University of Sheffield, Sheffield, England, S10 2RX, UK; 2 Neurosurgery, Royal Hallamshire Hospital, Sheffield, S10 2JF, UK; 3 Urology, Royal Hallamshire Hospital, Sheffield, S10 2JF, UK; 4 Karolinska Institut, Solnavägen, Solna, 171 77, Sweden; 5 Misvik Biology Ltd, Karjakatu, Turku, FI-20520, Finland; 6 Weston Park Hospital, Sheffield, S10 2SJ, UK

**Keywords:** Glioblastoma, ex vivo drug screening, functional precision medicine, glioma stem cells, cancer therapeutics, GliExP

## Abstract

With diminishing returns and high clinical failure rates from traditional preclinical and animal-based drug discovery strategies, more emphasis is being placed on alternative drug discovery platforms. *Ex vivo* approaches represent a departure from both more traditional preclinical animal-based models and clinical-based strategies and aim to address intra-tumoural and inter-patient variability at an earlier stage of drug discovery. Additionally, these approaches could also offer precise treatment stratification for patients within a week of tumour resection in order to direct tailored therapy. One tumour group that could significantly benefit from such *ex vivo* approaches are high-grade gliomas, which exhibit extensive heterogeneity, cellular plasticity and therapy-resistant glioma stem cell (GSC) niches. Historic use of murine-based preclinical models for these tumours has largely failed to generate new therapies, resulting in relatively stagnant and unacceptable survival rates of around 12-15 months post-diagnosis over the last 50 years. The near universal use of DNA damaging chemoradiotherapy after surgical resection within standard-of-care (SoC) therapy regimens provides an opportunity to improve current treatments if we can identify efficient drug combinations in preclinical models that better reflect the complex inter-/intra-tumour heterogeneity, GSC plasticity and inherent DNA damage resistance mechanisms. We have therefore developed and optimised a high-throughput *ex vivo* drug screening platform; GliExP, which maintains GSC populations using immediately dissociated fresh surgical tissue. As a proof-of-concept for GliExP, we have optimised SoC therapy responses and screened 30+ small molecule therapeutics and preclinical compounds against tumours from 18 different patients, including multi-region spatial heterogeneity sampling from several individual tumours. Our data therefore provides a strong basis to build upon GliExP to incorporate combination-based oncology therapeutics in tandem with SoC therapies as an important preclinical alternative to murine models (reduction and replacement) to triage experimental therapeutics for clinical translation and deliver rapid identification of effective treatment strategies for individual gliomas.

Research highlights **Scientific benefit(s)**

• Rapid analysis of drug responses within primary tumour cells
• Can be used with specific tumour niches post-surgery
• Drug responses can be tested alongside SoC therapies in patients as a clinical validation of the platform
• Facilitates potential drug repurposing
• Can be adapted for drug discovery/development
• Pre-printed drug plates can be tested against multiple different cancers once optimised
• Ideal model to investigate differential drug responses observed due to inter/intra tumour heterogeneity

**3Rs benefit(s)**

• Offers a significant reduction in use of animal-based preclinical models
• Replace the use of mouse orthotopic/cranial resection models currently rated as severe under ASPA Faster readouts of patient-specific drug responses
• Facilitates drug combination testing without the use of high numbers of animals

**Practical benefit(s)**

• Fairly easy to set up with standard commercially available equipment and image analysis software
• Facilitates preclinical research questions to be tested in a rapid time frame
• Scalability, many aspects could be readily automated
• Brings together scientific and clinical teams

**Current applications**

• Rapid identification of potential patient/tumour-specific therapeutic strategies for improved treatment responses
• Assessment of mono-therapeutic strategies, novel drug combinations and additions to current SoC treatments
• Currently in the academic setting but moving towards clinical trial/intervention-based platforms

**Potential applications**

• Implementation within a clinical trial/intervention setting to direct patient treatment
• Use within the commercial sector to assess novel therapeutics as well as drug discovery/development
• Expandable technology to potentially other solid cancers and/or those with a defined stem cell niche.

## Introduction

Brain tumours kill more children and adults under 40 than any other cancer, with high-grade glioblastomas being the most common cancers arising within the brain, contributing to ~200,000 deaths/year globally. ^1^^,^^2^ These tumours demonstrate large amounts of both inter- and intra-tumoural heterogeneity, ^3^^–^^6^ however our increasing understanding of the genetic diversity present within these tumours has not yet led to any tangible improvements in patient survival rates, which have stagnated over the last 40-50 years. ^3^^,^^4^^,^^6^^–^^10^ Additionally, these tumours harbour difficult-to-treat glioblastoma stem cell-like (GSC) subpopulations, ^11^^,^^12^ which possess regenerative potential and enhanced DNA repair capabilities. ^13^^–^^15^ As such, the majority of glioblastoma patients receive aggressive debulking surgical tumour resection followed by DNA-damaging radiotherapy and chemotherapy, which represents the current global standard-of-care (SoC) treatment for these currently incurable tumours. The mainstay chemotherapy is the DNA alkylating agent temozolomide (TMZ) ^16^ and around half of glioblastomas exhibit promoter methylation of the dealkylating enzyme MGMT. Although MGMT is an established predictive biomarker of TMZ effectiveness/clinical response, there is currently no superior alternative treatment for patients with an unmethylated MGMT promoter (MGMT ‘positive’), and survival for all patients receiving current SoC therapy remains around 12-15 months post-diagnosis. These current SoC treatment regimens have changed little in over a decade with high levels of innate and acquired treatment resistance giving rise to mean disease recurrence of around seven months, resulting in less than 10% of patients surviving more than five years. ^3^^,^^16^^–^^18^ Because of this, glioblastomas have been recognised globally as a cancer of unmet need that urgently requires new therapeutic interventions and approaches to improve treatment and patient survival. ^2^^,^^3^

Cancer studies using mice to provide personalised drug screening generally involve harsh tumour implantation, either orthotopically ( *i.e.* at the same anatomical site as the corresponding patient) or subcutaneously within the hind limb flank. ^19^ Since these studies usually assess response to various treatments, implanted tumours are permitted to grow to a pre-specified size/volume before starting treatment. Despite treatment, many mice will still experience symptomatic cancer progression, prior to the inevitable death/cull of all mouse participants. Consequently, such animal studies are classed as ‘moderate-severe’ depending on xenograft location, highlighting inherent animal distress, discomfort and non-survival. Murine-based preclinical models such as tumour flank models using established cell lines and patient-derived xenografts (flank and orthotopic) have traditionally been used for glioblastoma research because of their accessibility and because they share physiological characteristics and around 80% genetic homology to humans. ^20^ However, there is debate within the field as to whether or not the currently used plethora of murine preclinical models can accurately predict therapeutic responses in humans, ^3^ which is an important consideration given the high risk, cost and attrition rate associated with drug discovery/development. Such failure of these models is most likely due to several factors including the ability of tumour models to reflect the properties of post-surgical tumour cells ( *i.e.* residual disease), differences in pharmacokinetics, and the extreme cellular heterogeneity of gliomas not being recapitulated. ^21^ As such, oncology has the lowest possibility for success in drug development programmes. ^22^^,^^23^ The various advantages and disadvantages of commonly used glioblastoma models for preclinical drug screening including within drug development are discussed in more detail in our recent review article. ^19^ Additionally, using such models (as described in more detail below), a limited drug discovery study using patient-specific xenograft (PDX) models may require over 35 mouse avatars to provide individualised recommendations from a shortlist of five treatment strategies for one patient. Even using a single avatar model, typically at least 5-10 mice are required to test a single treatment. Consequently, time, cost, and ethical considerations limit the number of treatments that can feasibly be tested using PDX avatars for a single patient (usually less than 50). ^19^

There are three main types of *in vivo* models for primary brain tumours; chemically induced, genetically modified and xenograft (based either on established cell lines or more recently on patient-derived cells/tissue). ^24^ Traditional xenograft models involve the transplantation of human cancer cells into immunocompromised mice. Multiple established glioma cell lines such as U87, U251 and LN18 have been successfully xenografted. These models have advantages of high engraftment, reliable disease growth and progression, high growth rates with good reproducibility for investigating glioblastoma. Immortalised cell lines can be easily expanded *in vitro*, to yield large numbers of tumour cells for experimental use. ^25^ Despite this, cell line xenografts often do not resemble the complex clinical characteristics of the original tumour they are presumed to model. ^26^^,^^27^

More clinically relevant *in vivo* glioblastoma models include patient-derived xenografts (PDX), where surgically removed tumour biopsies or spheroids are implanted orthotopically or subcutaneously into immunocompromised mice. There are two main methods for establishing PDX models: the first involves the direct injection of biopsy tumour tissue, and the second is injection of cultured tumour spheres or cells harvested from monolayer cultures. Both methods can maintain the genetic and phenotypical features of the original patient tumour. Nevertheless, injecting biopsy tissue may be more clinically relevant as the architecture of original tissue is maintained, along with endothelium, extracellular matrix and resident macrophages. ^28^ However, one major caveat of PDX models is unsuccessful engraftment due to tumour sample size, aggressiveness, and low viability. PDX models are highly reliant on large tumour samples with good viability, to achieve successful engraftment, which is not always possible following surgical resection. This makes it increasingly difficult to model: lower grade gliomas such as oligodendrogliomas which proliferate much slower than high grade tumours; the invasive component of tumours which are more difficult to resect surgically and more likely to be left-behind; and small tumours in general, where less material is available for research. Once engraftment is successful it can take between 2-11 months for sufficient tumour growth, ^29^ which does not synergise with the rapid time frames required for guiding personalised therapy regimes given the rapid disease progression observed in aggressive cancers such as glioblastoma.

Early preclinical research in glioblastoma involved the use of both cell line and PDX xenograft models. Prior to TMZ approval as a SoC therapy, its use was investigated alone and alongside BCNU in three different cell line xenograft models: SNB-75, SF-295 and U251, with this study alone using a total of ~600 xenograft models. ^30^ Further preclinical investigations into TMZ utilised PDX xenograft models, where a total of ~280 murine models were used. ^31^ Another preclinical study investigated interstitial localised delivery of BCNU into controlled release polymers, which used a total of 344 9L gliosarcoma rat models, ^32^ which later led to the FDA approval of Carmustine wafers (Gliadel) that can be implanted into the surgical cavity following tumour removal as a complimentary treatment to TMZ; although it is not widely adopted due to marginal survival gains and a modified risk profile including increased wound breakdown and infection. ^33^ More recently, an ongoing clinical trial using the microtubule-destabilising drug lisavanbutin is showing potentially promising results (NCT02895360). ^19^ This is based on preclinical research showing the ability of the drug to penetrate the blood brain barrier (BBB) and increased survival fraction alone and alongside RT or RT/TMZ. This was determined using 14 different GBM PDX lines established as orthotopic xenografts, with each group containing 9-10 models giving a total count of up to 140 PDX models used. ^34^ As such, although various murine-based models can be an important preclinical validation step to promising clinical trials and potential subsequent clinical approval, more rapid and high-throughput clinically relevant preclinical drug screening approaches are required.

We therefore present here the development and optimisation of a high-throughput *ex vivo* drug screening platform for glioma stem cell populations: GliExP, that can be used to rapidly assess both preclinical and experiential therapeutics on an individual tumour basis, including intratumoural spatial heterogeneity and difficult-to-treat post-surgical/residual disease. By developing such an alternative *ex vivo* screening platform, we aim to reduce and replace a meaningful proportion of murine model use, which we estimate based on comprehensive analysis of the current global *ex vivo* solid tumour studies utilising murine avatars (see Table 2 within our recent review article) ^19^; this could amount to 2,000 mice per year not being used in preclinical high-grade glioma research, with the potential to impact on the current use of murine models for other solid tumours ( *e.g.* bladder, kidney etc). This is based on our analysis that on average, around 400 mice are used per study, and ~20,000 mice were used globally in this setting over the last five years. This estimate increases significantly when one considers our future plans to further develop GliExP to combine multiple therapeutics and in tandem with current SoC radiation and TMZ treatments.

## Methods

### Materials

**Table 1.** Materials and equipment used and supplier information.

Item	Company (product code)
**1,4-dithiothreitol (DTT)**	Sigma (DTT-RO)
**4',6-Diamidino-2-Phenylindole, Dihydrochloride (DAPI)**	Thermo Fisher Scientific (D1306)
**Accutase StemPro Cell Dissociation Reagent**	Invitrogen (A11105-01)
**ACK lysing buffer**	Gibco (A1049201)
**Advanced DMEM/F-12 (Dulbecco's Modified Eagle Medium/Ham's F-12)**	Invitrogen (12634028)
**Amphotericin B (Fungizone)**	Gibco (15290)
**B-27 Supplement (50x) Serum Free**	Invitrogen (17504-044)
**Benzonase Nuclease**	Novagen (70664-3)
**Bovine Serum Albumin (BSA)**	Sigma-Aldrich (A2153-100G)
**BreatheEasy Sealing Membrane**	Sigma (Z380059-1PAK)
**Cultrex Stem Cell Qualified RGF BME**	R&D Systems (3434-005-02)
**Dimethyl Sulfoxide (DMSO)**	Fisher Scientific (BP231-100)
**E1-ClipTip 16 Multichannel pipette**	Thermo Fisher Scientific
**EGF Recombinant Human Protein**	Invitrogen (PHG0313)
**Ethylenediaminetetraacetic acid (EDTA)**	Sigma (1233508)
**FGF Recombinant Human Protein**	Invitrogen (PHG0263)
**Foetal Calf Serum**	Lonza (BE12-60F4)
**Heparin Sodium Salt**	Sigma (H3393-10KU)
**High-Capacity RNA-to-cDNA Kit**	Thermo Fisher Scientific (4387406)
**L-Glutamine-200mM (100x)**	Invitrogen (25030081)
**Minimum Essential Medium (MEM)**	Invitrogen (10370-047)
**N-2 Supplement (100x) Serum Free**	Invitrogen (17502-048)
**NuPAGE 4-12% Bis-Tris Protein Gels –1.5mm, 10 well**	Thermo Fisher Scientific (NP0335BOX)
**NuPAGE 4-12% Bis-Tris Protein Gels –1.5mm, 15 well**	Thermo Fisher Scientific (NP0336BOX)
**NuPAGE LDS Sample Buffer (4X)**	Thermo Fisher Scientific (NP0007)
**NuPAGE MOPS SDS Running Buffer (20X)**	Thermo Fisher Scientific (NP0001)
**NuPAGE Transfer Buffer (20X)**	Thermo Fisher Scientific (NP0006)
**NuPAGE Tris-Acetate SDS Running Buffer (20X)**	Thermo Fisher Scientific (LA0041)
**Paraformaldehyde (PFA) – 4% solution in PBS**	Thermo Fisher Scientific (J60401)
**Penicillin-Streptomycin (10,000U/ml)**	Invitrogen (15140122)
**Perkin Elmer Cell Carrier Ultra 384-well**	Perkin Elmer (6057302)
**Phosphatase Inhibitor Tablets (PhosSTOP)**	Sigma (4906845001)
**PierceECL Western Blotting Substrate**	Thermo Fisher Scientific (32106)
**Prolong Gold Antifade Mountant**	Thermo Fisher Scientific (P36930)
**Protease Inhibitor Cocktail (cOmplete ULTRA Tablets Mini *EASYpack*)**	Sigma (5892970001)
**RNeasy Mini Kit**	Qiagen (74104)
**Sodium chloride**	Sigma (S7653)
**TaqMan Universal PCR Master Mix (No AmpErase UNG)**	Applied Biosystems (4324018)
**ThermoMixer C**	Eppendorf (5382000031)
**Tris Hydrochloride**	Sigma (10812846001)
**Triton X-100**	Sigma-Aldrich
**Trypan Blue**	Sigma (T8154-20ML)
**MicroAmp Optical Adhesive Film**	Applied Biosystems (4311971)
**Sterile metal ruler**	Northbrook (B08G1RD1Y7)
**Nunc Cell Scrapers**	Thermo Fisher Scientific (179707PK)
**Nitrocellulose Membrane (Protran)**	VWR (10600010)

### Maintenance of GSC culture conditions

Preparation of GSC ‘stem’ culture media:

Here we describe the procedure used to prepare 500 mL of GSC media used to maintain stem-like cell populations. Reagents used are listed in Table 1.

1. Under sterile conditions add 1% B27, 0.5% N2, 1% L-glutamine, 1% Penicillin-streptomycin, 0.1% amphotericin B, 4 ug/mL heparin to 500mL bottle of advanced DMEM F12 media.
2. Manually pipette solution up and down to ensure components are fully mixed.
3. Prepare stocks of 0.1 mg/mL epidermal growth factor (EGF) and 0.1 mg/mL fibroblast growth factor (FGF) by dissolving in sterile PBS, aliquot into 20-40 μL stocks and store at -20°C until further use.
4. When media is required, transfer an appropriate volume into upright separate sterile T75 flasks prewarmed to 37°C in an incubator.
5. Add 20 ng/mL EGF and FGF to media, this is made fresh daily to avoid degradation of growth factors.

Preparation of GSC ‘bulk’ culture media:

Here we describe the procedure used to prepare 500 mL of differentiating ‘bulk’ media, the added serum induces differentiation of GSC cultures.

1. Under sterile conditions add 10% FCS, 1% L-glutamine, 1% penicillin-streptomycin and 0.1% amphotericin B to a 500 mL bottle of MEM media.
2. Manually pipette solution up and down to ensure components are fully mixed.
3. No extra growth factors required so can use media directly from bottle stock.

Protocol for coating plasticware with basement membrane:

Here we describe the procedures used to coat plasticware with basement membrane extract (BME) (Cultrex), this allows GSC populations to grow as adherent monolayers. Concentrations and volumes used are listed in Table 2. To avoid any batch-to-batch variation of this product, sufficient volumes were purchased to cover the entirety of the project.

1. Add BME Cultrex (5-10 mL) stock into ice bucket and thaw overnight in the fridge at 4°C.
2. Under sterile conditions aliquot Cultrex into 1mL stocks and store at -80°C until further use (work on ice or work rapidly to prevent solidification of BME).
3. When required thaw a Cultrex aliquot overnight as per step one.
4. Dilute 1 mL of Cultrex with 39 mL (1:40) cold advanced DMEM F12 (without supplements and growth factors) gently pipette up and down to mix with a pre-chilled pipette.
5. At room temperature, pipette onto plasticware at appropriate volumes (see Table 2) then gently tilt in all directions to ensure even coverage of liquid.
6. Place coated plasticware into incubator set at 37°C for 30 minutes to polymerise.
7. Gently tilt plasticware and remove excess media using a pipette, ensure flasks lids are securely fastened and 6 well plates are taped shut.
8. Cultrex-coated plasticware can be stored in the fridge at 4°C for up to 14 days.

**Table 2.** Cultrex coating plasticware.

Plasticware	Concentration cultrex	Volume cultrex-media added (μL)
75 cm Tissue Culture Flasks	1:40	2500/flask
25 cm Tissue Culture Flasks	1:40	1500/flask
6-well Treated Plates	1:40	500/well
384-well Perkin Elmer Drug Plates	1:20	5/well

Protocol for coating 384-well drug plates with basement membrane:

Here we describe the procedures used to coat pre-treated 384 drug plates with basement membrane extract (BME; Cultrex™).

1. Add 1mL BME aliquot into ice bucket and thaw overnight in the fridge at 4°C.
2. Dilute 1mL of BME with 19 mL (1:20) cold advanced DMEM F12 (without supplements and growth factors) gently pipette up and down to mix.

*Note – BME 1:20 dilution used for drug plate coating to account for dilution when added the wells pre-coated with 5 μl drug.*

3. Pour diluted BME into a 50 mL reagent reservoir.
4. Using an automated 16 channel E1-ClipTip and pre-chilled tip, pipette 5 μL per well into 384 well drug plate.
5. To ensure even coverage of BME centrifuge plate for 30 seconds at 800 RCF at room temperature.
6. Place coated drug plate into incubator set at 37°C for 30 minutes to polymerise.
7. Once polymerised drug plates were used immediately to avoid degradation of drug.

### Drug plate printing

Here describes the protocol used for the 384-well drug plate printing. Drug compounds can be purchased as either pre diluted liquids or solids and diluted in DMSO (or water for platinum-based compounds such as cisplatin) to stock working concentrations of 5-10 mM and stored in -80°C. All compounds used in this study are listed in Table 3 with their concentrations.

**Figure 1.**
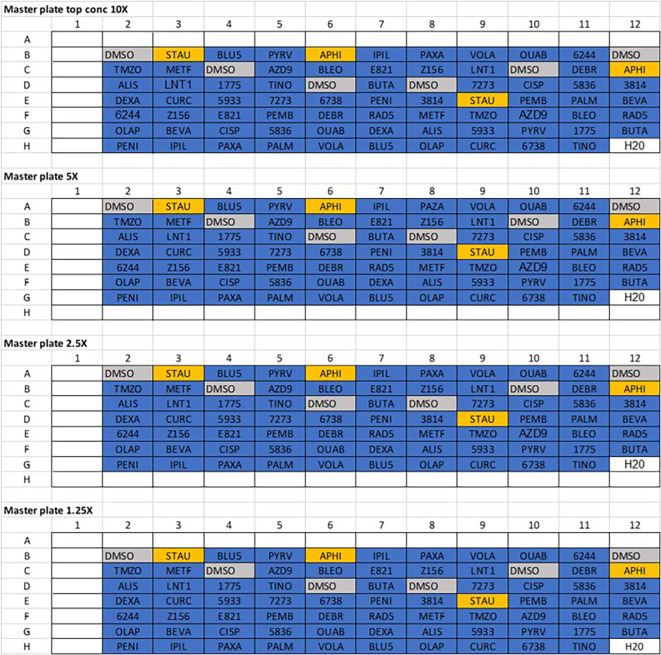
Master 96-well plate layouts. Initially, compounds were diluted to ××10 the top concentration to be used and added to their corresponding wells on the ×10 master plate. Drugs from this plate were then serially diluted down 1:2 into ×5, ×2.5 and ×1.25 master plates. Vehicle control (DMSO) and positive controls (Staurosporine and aphidicoline) were added to the plate independently at single concentrations.

1. Store compounds purchased as liquid according to manufacturer’s instructions, to avoid any extra freeze thaw cycles thaw and aliquot as appropriate on day of drug plate printing.
2. Dilute all compounds to 10X their top concentration (see Figure 1 below) and add to a 96-well master plate in duplicate, ensure duplicates are not in a similar region of the plate (to avoid any plate effects).
3. This plate layout can be designed and known by the user prior to any subsequent analysis. Only use 77 wells, leaving row A and column 1 empty.

**Table 3.** Drug compounds and concentrations used on 384 well drug plate.

Drug	Supplier	Concentration (μM)			
		P1	P2	P3	P4
Stausporine	Stratech (Selleckchem)	5	5	5	5
Aphidicolin	SantaCruz	2	2	2	2
Alisertib	Stratech (Selleckchem)	5	2.5	1.25	0.625
AX15836	Medchem Express	20	10	5	2.5
AZD0156	Stratech (Selleckchem)	5	2.5	1.25	0.625
AZD1775(Adavosertib MK-1775)	Stratech (Selleckchem)	5	2.5	1.25	0.625
AZD6244 (Selumetinib)	Stratech (Selleckchem)	2	1	0.5	0.25
AZD6738	Stratech (Selleckchem)	5	2.5	1.25	0.625
AZD9291 (Osimertinib)	Stratech (Selleckchem)	5	2.5	1.25	0.625
Bevacizumab	Stratech (Selleckchem)	25	12.5	6.25	3.125
Bleomycin	Stratech (Selleckchem)	20	10	5	2.5
Fisogatinib (BLU-554)	Stratech (Selleckchem)	10	5	2.5	1.25
Butamben	Stratech (Selleckchem)	20	10	5	2.5
Cisplatin	Stratech (Selleckchem)	20	10	5	2.5
Curcumin	Stratech (Selleckchem)	10	5	2.5	1.25
D-Penicillamine	Stratech (Selleckchem)	20	10	5	2.5
Dabrafenib	Stratech (Selleckchem)	25	12.5	6.25	3.125
Dexamethasone	Stratech (Selleckchem)	5	2.5	1.25	0.625
Ipilimumab	Stratech (Selleckchem)	25	12.5	6.25	3.125
KU55933	Stratech (Selleckchem)	10	5	2.5	1.25
LNT 1	Tocris Bioscience	5	2.5	1.25	0.625
M3814 (Nedisertib)	Stratech (Selleckchem)	5	2.5	1.25	0.625
Metformin	Stratech (Selleckchem)	50	25	12.5	6.25
Olaparib (AZD2281)	Stratech (Selleckchem)	10	5	2.5	1.25
Ouabain	Stratech (Selleckchem)	2	1	0.5	0.25
Palmoic acid	Stratech (Selleckchem)	10	5	2.5	1.25
Paxalisib	Stratech (Selleckchem)	5	2.5	1.25	0.625
PDD00017273	Stratech (Selleckchem)	5	2.5	1.25	0.625
Pembrolizumab	Stratech (Selleckchem)	25	12.5	6.25	3.125
Pyrvinium Pamoate	Stratech (Selleckchem)	10	5	2.5	1.25
RAD51 Inhibitor B02	Tocris Bioscience	10	5	2.5	1.25
Temozolomide	Stratech (Selleckchem)	500	250	125	62.5
Tinostamustine	Stratech (Selleckchem)	5	2.5	1.25	0.625
VE-821	Stratech (Selleckchem)	5	2.5	1.25	0.625
Volasertib	Stratech (Selleckchem)	2	1	0.5	0.25

*Note – Row A and column 1 are left empty as these will correspond to the outer wells of the final 384 drug plate, which are left empty due to plate evaporation effects.*

4. Serially dilute this master plate into three separate 96 well master plates using a 1:2 dilution, to give four plates total with 10X, 5X, 2.5X and 1.25X dilution factor examples shown in Figure 1.
5. Following this, add negative vehicle control; DMSO (n=24) and positive controls; staurosporine (n=8), and aphidicolin (n=8) independently. If using water soluble drugs such as cisplatin water should also be added as negative vehicle control.
6. Rotate the 5X and 1.25X plates 180 degrees prior to transfering to 384 well format to ensure all four dilutions are not next to each other. Create the master plate layout ensuring all drug repeats, dilutions and controls are spread across the innermost 308 wells of a 384-well plate (Perkin Elmer 384 Cell Carrier Ultra) this avoid any bias resulting from edge evaporation effects or well positioning.
7. Using a ThermoFisher Scientific Matrix Platemate Plus, dispense 5μL from the four master plates onto the allocated 384-well plate well, in addition to any vehicle control wells. Once the 384-well plates are printed, briefly centrifuge for 30 seconds at 800 RCF and seal with a non-permeable cover and immediately store at -80 ^o^C.

### Patient recruitment and sample transfer

Fresh treatment-naïve glioblastomas were collected from patients who provided informed consent undergoing surgery at Sheffield Teaching Hospitals NHS Foundation Trust (Ethical approval: Yorkshire & The Humber – Leeds East REC (11-YH-0319/STH15598). Fresh glioblastoma tissue surplus to histological requirements resected by the operating surgeon was collected intraoperatively whilst surgery was ongoing, pseudonymised/assigned a unique sample identifier and then rapidly transferred to the *ex vivo* laboratory within <30 minutes within a dry sterile specimen pot (NHS) at room temperature. Tumour samples were taken from both male and female patients across a range of ages within the parameters associated with the prevalence for this disease and the local gender/age consenting at Sheffield’s Royal Hallamshire Hospital and anonymised to the *ex vivo* screening team.

### GliExP sample processing and culture

Here we describe the protocol used for the dissociation and seeding of tissue specimens onto pre-coated 384-well drug plates.

1. Prior to collection of tissue ensure to pre-warm stem media, Accutase and PBS to 37°C.
2. Place tumour sample into 50-100 mm petri dishes depending on the tissue size and wash thoroughly with pre warmed phosphate buffered saline (PBS)
3. Aspirate all excess PBS, measure tissue specimens with a sterile metal ruler, divide any tissues larger than 20×20×20 mm into half or a third using a scalpel into separate petri dishes in order to model intratumoural heterogeneity ( *e.g.* GBM1 sample A, B, C).
4. Further divide these sections into smaller 2×2×2 mm regions prior to dissociation. Add 10-20 mL of pre-warmed stem media, without growth factors to immerse sample.
5. Mechanically dissociate the sample using forceps and a p1000 pipette tip using pulling motions. For more calcified solid tumours dissociate using a scalpel.
6. For smaller samples not modelling heterogeneity carefully divide the total tissue homogenate equally using a plastic Pasteur pipette into 4-6 15 mL falcon tubes to give a maximum of ~0.5 pellet of tissue per tube.

*Tip – If the tissue homogenate is too large to be picked up with Pasteur pipette, repeat mechanical dissociation. Be careful when using pipette as tissue can suck up into bulb.*

7. Split larger tissues modelling intratumoural heterogeneity into appropriately labelled 15mL falcon tubes ( *e.g.* Sample A, B, C) using ~4-6 tubes per sample.

*Tip – It is possible to derive smaller samples with 1-2 ~0.5 mL pellets, however samples screened in this study are usually in excess, therefore we find it important to dissociate the majority of tissue to give accurate representation of cells in entire the tumour.*

8. Centrifuge the 15mL tubes at ~180 RCF for 3 minutes at room temperature, after which carefully remove the supernatant. If the residual pellet contains a large red blood cell (RBC) layer, then add pre-warmed PBS to each tube, agitate using a Pasteur pipette and leave to settle for 10 mins.
9. Centrifuge tubes at 180 relative centrifugal force (RCF) for 30 seconds at room temperature, carefully aspirate supernatant with plastic Pasteur pipette.
10. Add 3mL Accutase warmed to 37°C to each individual tube and the pipette suspension up and down to provide further disaggregation prior to agitated incubation using ThermoMixer C (Eppendorf) set at 37°C 750 rpm for 30 minutes. Repeat this enzymatic dissociation step until cells are well dissociated with the majority as single cells (typically 45-60 minutes). Check dissociation under a brightfield microscope using glass coverslips.
11. Add 7 mL stem cell media to each falcon tube and mixed well using a 10/5 mL stripette. Centrifuge cell suspensions at ~180 RCF at room temperature, then remove supernatant.
12. Resuspend pellets in 3 mL stem media, mix well then filter through a 70 μM cell strainer into a 50 mL falcon tube.
13. At room temperature, divide cell solutions equally into 15 mL falcon tubes (one per every 2×2 mm tissue sample) and then centrifuge for 5 minutes at ~180 RCF, if a RBC layer is still present on the pellet, add 5 mL of ACK lysing buffer per tube, mix well and leave for 5 minutes before further centrifugation at ~180 RCF for 5 minutes.
14. Following aspiration of the supernatant, resuspend cells in 5 mL PBS, mix well and centrifuge again at ~180 RCF for 5 minutes at room temperature, aspirate the supernatant aspirated and resuspended pellet in stem media including growth factors (EGF and FGF).
15. Count and viability test the cells with trypan blue using Cellometer Mini (Nexcelom Bioscience) cell counter.

*Note – Viability was typically between 20-40% for these methods.*

16. Using the live cell count, dilute cells in stem media containing growth factors into a 50mL falcon tube to give an appropriate density (usually 5-10k cells per well in 384 well).
17. Using the E1-clip 16 channel pipette and a 50 mL reagent reservoir dispense 40 μL of the resulting suspension to all but the outer wells of the pre-loaded drug ECM coated drug plates, making the total volume 50 μL.
18. Fill the empty outer wells of the plate with 100 μL media before sealing the plate with “breathe easy” sealing membrane to help with any evaporation effects.
19. Place drug plates within an incubator (37°C in 5% CO _2_) for a 4–8-day incubation period, which was set based on previous publications using similar techniques. ^35^^,^^36^

*Note – End users should test different incubation periods to optimise appropriate incubation times for their specific needs/cell populations.*

20. Label samples with unique identifier *e.g.* p-GBM1a, prefix ‘p’ represents primary dissociated tissue
21. For follow up experiments it is important to culture primary samples in tandem with drug screening, this is described in detail in the following section.

Here we describe the protocol used for the culturing of GliExP samples for relevant follow up experiments:

1. Place any residual cell solution into an upright T25 flask (non-adherent conditions) and place into an incubator (37°C in 5% CO _2_) 24-48 hours to facilitate the formation of primary neurospheres.
2. Collect and transfer into BME-coated flasks to form primary monolayer
3. Once adhered gently wash with PBS to remove residual RBCs and any cell debris
4. Replace the stem media (with growth factors) every 3 days
5. Once confluent transfer cells to two T25 flasks or one T75 flask to generate a secondary monolayer. Continue to expand cells and cryopreserve aliquots for long-term stocks and future experiments as needed
6. Label flasks and cryovials with appropriate GBM identifier (without ‘p’ prefix as this represents freshly dissociated tissue rather than established GSC models)

**Figure 2.**
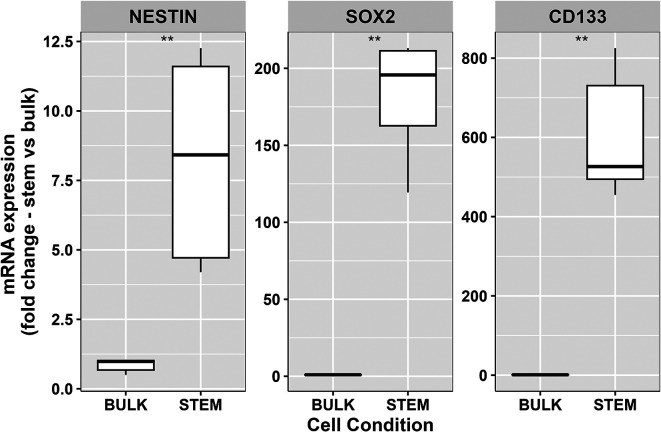
RT-PCR analysis of mRNA expression of stem cell markers nestin, SOX2 and CD133 within two model growth conditions ‘stem’ and ‘bulk’. Expression levels of all three GSC markers mRNA were higher within passage matched GSC populations compared to their bulk equivalent cells. Data represents the 25 ^th^ and 75 ^th^ percent quartiles (box) median (middle line). The lower and upper whiskers show the smallest and largest values within 1.5x of the interquartile range below the respective 25 ^th^ and 75 ^th^ percentile. Data shown is derived from two independent biological repeats from separate passages of the same patient derived cell line, with each repeat including three technical replicates for each marker condition. Statistical significance was calculated using the Mann-Whitney-U test (**P<0.002).

*Note – for the purpose of this paper unless the ‘p’ prefix is used, the GSCs are derived from matched dissociated tissue. Furthermore, although well-established in our laboratory, to confirm that our GSC growth media appropriately maintained GSC niches within the 384-well format, we compared known GSC marker expression in matched GSC CX18 cell line populations grown in bulk and stem enrichment media and that GSC plasticity was maintained* ( Figures 2 *and* 3 *respectively*).

**Figure 3.**
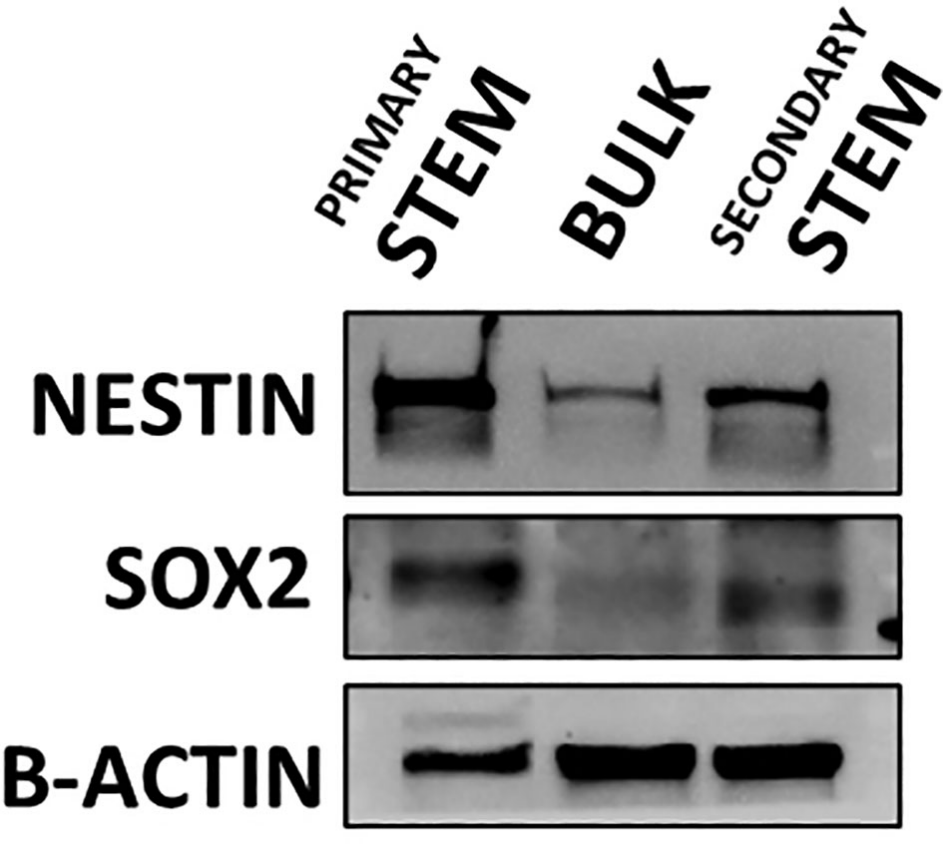
Western blot detecting the expression of stem cell markers nestin and Sox2 within undifferentiated (primary stem), differentiated (bulk) and de-differentiated (secondary stem) cultured CX18 GSCs. Serum differentiated CX18 GSCs show the ability to revert to a de-differentiated state re-expressing stem markers SOX2 and Nestin, when bulk cells are cultured back in stem growth conditions, thus demonstrating and confirming GSC plasticity.

### Immunofluorescent staining

*Staining of drug plates*

Here we describe the protocol used for the immunofluorescent staining of 384-well drug plates for microscopy imaging to evaluate treatment response. These fixing, washing and staining steps have been optimised to reduce the number of tumour cells being washed away.

1. Following a 4-8 day incubation, place drug plates into a class II laminar flow hood and remove breathable membranes.
2. Using a E1-clip 16 channel pipette and a 50mL reagent reservoir add 40 μL cold 4% paraformaldehyde (PFA) containing 0.6% Triton-X to each well then incubate at room temperature for 30 minutes before aspirating fully.
3. Using a different reagent reservoir add and remove 30μL of PBS to wash each well.

*Tip – At this stage it is possible to add another 30μL of PBS and store the plate at 4°C for up to 48hrs or proceed directly with staining steps.*

4. If proceeding with staining make up antibody solutions using PBS, Tween-20 (0.05%) and bovine serum albumin (3%), all antibodies used, concentrations and their suppliers are listed in Table 4. Use DAPI as a marker for cell nuclei at a concentration of 1 μg/mL.

**Table 4.** Antibodies used for immunofluorescence.

Antigen	Raised in	Conjugated	Application and dilution	RRID	Company
CD133 (Prominin)	Rabbit	Unconjugated	IF 1:250	AB_2847920	Abcam (ab216323)
Nestin	Mouse	Alexa Fluor 488	IF 1:100	AB_627994	Santa Cruz (sc-23927)
SOX2	Mouse	Alexa Fluor 546	IF 1:75	AB_10842165	Santa Cruz (sc-365823)
Vimentin	Mouse	Alexa Fluor 546	IF 1:400	AB_10917747	Santa Cruz (sc-373717)
Anti-Rabbit Secondary antibody	Goat	Alexa Fluor 488	IF 1:500	AB_143165	Life Technologies (A-11008)

*Tip – where possible implement conjugated antibodies to reduce washing steps and retain cell population on plates.*

5. Add 10 μL of antibody solution to each well (ensure the PBS has been removed). Cover plates with foil and incubate overnight at 4°C.
6. The following day wash cells and resuspended in 30 μL PBS ready for microscopy analysis.

*Tip – If not proceeding with microscopy immediately, plates can be wrapped in foil and stored in the fridge at 4°C. It is recommended to image the plates within a week of staining to avoid degradation of antibodies.*

7. If using unconjugated antibodies such as CD133, the primary antibody and DAPI should be added as described in step 5. On the following day, wash wells with PBS, then add appropriate secondary antibody made up in PBS (see Table 4 for dilutions) at room temperature for 1 hour. Wash cells and add 30μL PBS ready for microscopy analysis or store in the fridge at 4°C.

### Optimisation of GSC markers by immunofluorescence

Glioblastoma stem and bulk cells from early passage primary tumour samples (Rominiyi *et al*., under revision) were seeded at a density of 3×10 ^5^ cells per well onto BME-coated coverslips inside a six-well tissue culture dish. Cells were then incubated at 37°C in 5% CO _2_ for 48 hours without treatment. The cells were then fixed and permeabilised using cold 4% PFA and 0.6% Triton X-100 (500 μL) for 10 minutes, followed by three PBS washes (5 minutes each). Cells were then blocked for 1 hour at room temperature in PBS containing BSA (3%). Conjugated and primary antibodies used were diluted in PBS containing Tween (0.05%) and bovine serum albumin (3%), 500μl of required antibody was added to each well at appropriate concentration ( Table 4) and incubated overnight at 4°C in the dark. The following day the primary/conjugated antibodies were then aspirated before 3 additional washes with PBS. Next 500 μL of DAPI and/or secondary antibody was added for one hour at room temp on a gentle shaking platform in the dark to prevent photobleaching, the antibodies were aspirated and washed with PBS (5 minutes each). Following the final wash, coverslips were mounted onto labelled microscope slides using ProLong Gold Antifade Mountant (Thermo Scientific). Slides were then stored in the dark to drug overnight, before being stored in the fridge at 4°C. Antibodies for GSC markers were CD133, Nestin and SOX2, with Vimentin used as a marker of differentiation and DAPI used to identify cell nuclei.

### Microscopy imaging

*Drug plate imaging*

Here describes the protocol used for the microscopy imaging of sample drug plates, all plates in this study were imaged using a Cell Discoverer 7 microscope using Zen Blue 3.1 software.

1. If drug plates have been stored in the fridge, remove and leave at room temperature for half an hour to remove condensation.
2. Wipe bottom of 384-well plate clean with 70% industrial methylated spirit (IMS) and lint free tissue and insert onto microscope stage
3. Select the appropriate channels based on the excitation of the conjugated antibodies used, along with brightfield and DAPI as the reference channel.
4. Select the lens, this study used a 10× magnification using the 20×, 0.5 lens.
5. Calibrate the software using the brightfield channel as a reference to set the peripheral wells and ensure the plate is positioned correctly.
6. Select the tile strategy for plate, which by default set a non-overlapping five-tile strategy chosen by software, however on plates with reduced numbers of cells (common with freshly dissociated cells) increase this number to 9-12 tiles per well to increase the imaged area per well (5 tile = 28.6%, 9 tile = 51.5%, 12 = 68.7% of individual 384 well area).
7. Set up the focus using DAPI as the reference channel, any of the additional antibody markers from this reference adding any offset values to the appropriate channels tab.
8. Complete image capture by running experiment, this usually takes between 2-4 hours depending on the number of channels and density of cells. The default order at which the images are captured is left to right, top to bottom per well, and a serpentine pattern for the 384 well plate, left to right, down, right to left, down, and repeat.

### Coverslip imaging

Imaging of microscope slides was performed on an LSM-980 confocal microscope all images were taken using the Photometrics sCMOS Prime BSI imaging camera using 40x objective. Images were captured using the Zeiss Blue (3.0) software package with all settings (laser power, digital gain, digital offset) kept constant between experimental conditions and repeated measures.

### Image analysis

**Figure 4.**
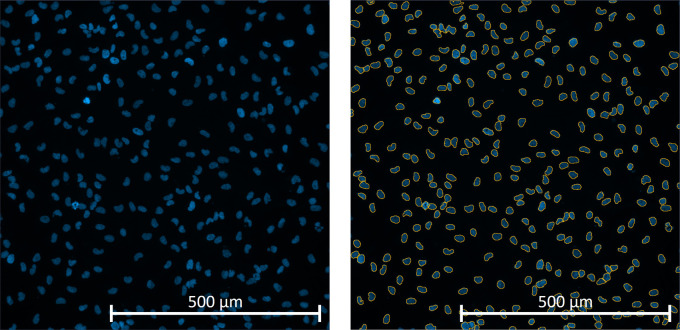
Example of how the Zen Blue software images were obtained using the Cell Discoverer 7 10x magnification. Primary glioblastoma positively DAPI stained nuclei were identified as objects by the algorithm for scoring purposes (yellow outlines).

All image analysis was performed using Zeiss Zen Blue 3.5 analysis software. Using the processing tab, an image subset from the initial image file was extracted selecting approximately 30 individual tiles, which included a selection of wells from positive, Staurosporine and negative DMSO control wells along with any wells containing significant debris or artifacts. This subset was then selected to train the software using the Zen Intellesis machine learning module, which allows segmentation of images based on the user training distinguishing between object and background. Objects (DAPI positive nuclei) were manually labelled using the labelling options tab with the brush option, background including artifacts were labelled the same way. The setting dropdown box was set to 50 features, as this enables Zen Intellesis machine learning module to full access the computer GPU, reducing the time taken to fully analyse the images ( Figure 4). The supervised learning had to be done independently for each sample as every sample is very heterogeneous, therefore the model could not be transferred between different GBM samples. Once the Intellesis training model was completed a sample specific image analysis program was then created.

### RStudio analysis

Plate layout templates were designed which contained compound name, concentration and well number it corresponded to. Using RStudio software version 4.2.2 the output CSV files that were produced by Zen Blue were initially concatenated using the summarise function by dplyr to obtain a DAPI frequency count per well. This file was then merged with the plate layout template by well number, so that each well corresponded to DAPI frequency, drug and concentration. Any data visualisation drug response plots were produced using the ggplot2 package. In order to obtain cell metrics such as LD _50_ values and AUC, a package called GRmetrics was used. GRmetrics files for each sample was then imported back into RStudio to generate a heatmap representing AUC values. This was performed using the pheatmap package.

### Quantitative PCR

For RNA extraction, Bulk/stem cells were seeded into Cultrex coated 6-well plates and incubated until 70% confluent before harvesting. Qiagen RNeasy Mini Kits were used to extract RNA. Initially, media was aspirated from cultured cells and cells were washed twice with PBS. PBS was then aspirated and 350 μL RLT buffer was added to each well and cells were then dislodged using a cell scraper and transferred to labelled QIAshredder columns before being centrifuged at 8000 RCF for 2 minutes. The purple columns were discarded and 350 μl of 70% ethanol was added to the supernatant, this solution was then transferred to a RNeasy Minispin column and centrifuged for 15 seconds. The flow through was discarded and 700 μl RW1 buffer was added to each column and centrifuged for another 15 seconds at 800 RCF. This process was then repeated again twice but with 500 μl RPE buffer, the first instance centrifuged for 15 seconds and the second for 2 minutes, discarding the flow through for each step. Excess ethanol was then removed by centrifuging the column for 1 minute, before adding 50 μl of RNase free water and centrifuging again for another 1 minute to elute the RNA. Total RNA was then quantified for each sample using Nanodrop 200 spectrophotometer, which quantifies absorbance at wavelength at 260 nm to establish the concentration of RNA in each sample, and the ratio of sample absorbance at 260/280 was used to confirm purity (absence of contaminants such as protein or phenol, which absorb strongly at/near 280 nm) with ratios of ~2 deemed to represent ‘pure’ RNA. After quantification RNA samples were either used immediately or stored at -80°C. RNA samples were reverse transcribed using High-Capacity RNA-to-DNA Kit (Applied Biosystems). The RT reaction was prepared on ice in order to obtain a 30 μl sample of cDNA for downstream qPCR. Volumes of sample equivalent to 1μg of RNA were added to reagents in the kit after 15 μl buffer and 3 μl of enzyme and made up to 30 μL using RNase-free water. Samples were then reverse transcribed using a Biorad thermal cycler T100 using cycling parameters 37°C for 1 hour, 95°C 5 minutes to convert RNA into cDNA. Each resulting cDNA sample was analysed in triplicate for each individual probe within a 384-well PCR plate with GAPDH used as a control ‘housekeeping’ gene for each sample. Each reaction consisted of: 2 μL cDNA, 5 μL TaqMan Universal PCR Mastermix, 2.5 μL ddH _2_O and 0.5 μL of probes (provided in Table 5). The plate was sealed using optical adhesive film (MicroAmp) and loaded onto a 7900HT Fast Real-Time PCR System to perform quantitative PCR. The system was set to report 18S FAM with repeats of 40 cycles. Cycling conditions involved an initial 95°C hold for 10 minutes followed by 40 cycles of 15 second 95°C denature step and finally 60°C for 1 minute in order to anneal and extend. Double delta Ct (2-ΔΔCt) analysis was used to determine relative gene expression using an average Ct value from the triplicate runs. For example, to detect differences in expression between primary, patient derived glioblastoma stem cells grown in stem conditions and bulk differentiated cells: ΔCt = Mean Control (GAPDH) Probe Ct – Mean Gene Probe Ct, and ΔCt Expression = 2-ΔCt. Then, to calculate fold-change expression in GSCs relative to bulk cells: ΔΔCt = ΔCt Expression (Stem)/ΔCt Expression (Bulk).

**Table 5.** TaqMangene expression probes for quantitative PCR.

Gene	Assay ID	Fluorescent reporter dye	Company
GAPDH	Hs02758991_g1	FAM	ThermoFisher
CD133	Hs01009259_m1	FAM	ThermoFisher
NESTIN	Hs04187831_g1	FAM	ThermoFisher
SOX2	Hs01053049_s1	FAM	ThermoFisher

### Immunoblotting

For immunoblotting glioblastoma (bulk/stem) cells were seeded into Cultrex-coated 6-well plates and incubated at 37°C in 5% CO _2_ for 48 hours without any treatments. Before media removal, cells were washed twice in ice-cold PBS. Cells were then lysed with the addition of 100 μL lysis buffer (20 mM Tris-HCl pH 7.5, 150 mM NaCl, 1% Triton X-100, 1 mM DTT and 1 mM EDTA supplemented with 50 U/μL benzonase (Novagen), protease and phosphatase inhibitors (Sigma). Cells were immediately harvested using a cell scraper and transferred to labelled Eppendorf tubes on ice. Each sample was then vortexed for 10 seconds before being placed on ice for 15 minutes. Samples were vortexed again following a final 15-minute incubation on ice and one final vortex. Samples were then centrifuged at 14,000 × g for 15 min at 4°C. Gel electrophoresis was performed using NuPage system (Invitrogen). Samples were resolved on 4-12% Bis-Tris gels in MOPS buffer, transferred to Protran nitrocellulose membranes (0.1 μm pore size), which were then probed for proteins of interest using antibodies diluted in 5% BSA (details of antibodies shown in Table 6 below).

**Table 6.** Antibodies used for immunoblotting.

Antigen	Raised in	RRID	Application and dilution	Company
Nestin	Mouse	AB_305313	WB 1:1000	Abcam (ab6142)
SOX2	Mouse	AB_10842165	WB 1:500	Santa Cruz sc-365823)
b-Actin	Mouse	AB_626632	WB 1:2000	Santa Cruz (sc-47778)

### Statistical analyses

Shapiro-Wilk normality tests were performed to assess the datasets prior to statistical analysis to decide the correct tests to perform. In order to confirm differences between two groups Mann-Whiney U-test was performed on non-parametric datasets or Students t-test on parametric datasets, p-values less than 0.05 would determine whether the differences in results were significantly different. All normality tests and statistical analysis was performed using R package *stats* version 4.2.2. As the current study is not comparing *ex vivo* drug responses to any clinical indications, normal power calculation or simulation studies were not used to determine the sample size at this time.

## Results

### Optimisation of plating order and SoC therapy responses to support a high-throughput drug screening platform for primary GSCs

**Figure 5.**
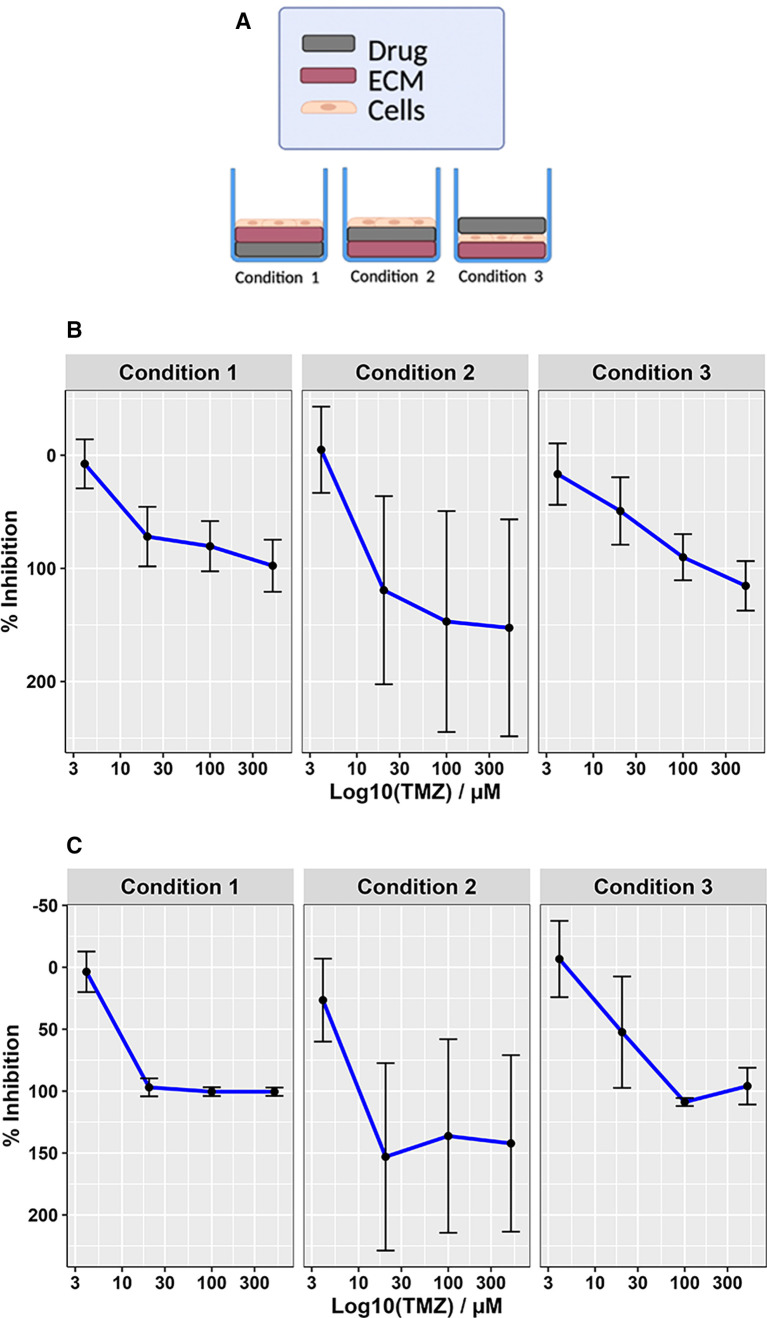
Optimisation of plating conditions for high-throughput GSC drug screening. A: Diagrammatic representation of the three different drug, cell and ECM alternations tested to ensure drug efficacy within the screening platform. B: TMZ dose response curves for CX18 GSCs following 4-day drug incubation using the different plating conditions. C: Same as (B) but following an 8-day TMZ incubation/growth period. For both datasets, mean data derived from 3 independent biological repeat experiments is shown with their respective SDs. Condition 1 average LD _50_ = 18.95 ± 3.45 μM, condition 2; 16.28 ± 6.22 μM, and condition 3; 56.60 ± 50.15 μM for 4 days TMZ incubation/growth. For 8 days, Condition 1 average LD _50_ = 9.61 ± 4.9 μM, condition 2; 6.07 ± 0.87 μM, and condition; 23.04 ± 4.04 μM.

Glioblastoma cultures grow as neurospheres unless a BME is provided, giving structural support for the cells, thus providing a dense layer of extracellular matrix (ECM) to allow them grow as adherent monolayers. For the purpose of high throughput screening, monolayer growth is attractive as it will facilitate a rapid examination of cell-drug interactions using automated high-content microscopy. However, the requirement to pre-coat plates with a basement membrane that can only be stored for a couple of weeks once used, introduces a level of complexity compared with standard 2D established cell line-based monolayer drug screening. To keep the process as streamlined as possible, drug plates would ideally be pre-printed, stored at -80°C, then on the day of surgery the plates thawed and coated in ECM ready for GSC sample seeding directly from the clinic. Using the glioblastoma SoC drug Temozolomide (TMZ) we therefore determined if the order by which the ECM, compounds and cells affected drug efficacy ( Figure 5), using primary GSCs derived from a patient tumour. The LD _50_ values for TMZ in Conditions 1 and 2 were consistent within both the four-day and eight-day incubation periods, highlighting that when TMZ was seeded underneath the ECM it still has the same efficacy as if it was seeded above. However, within both the four-day and eight-day incubation Condition 2 showed much higher variation for percentage inhibition within repeats, suggesting this method of drugging may lead to discrepancies between repeated measures. Additionally, Condition 3 showed an increased LD _50_ compared to the other 2 conditions, which may be a consequence of higher cell adherence and cell cycling, by allowing the cells to adhere to basement membrane prior to drugging. Despite Condition 3 being the most representative model of patient tumour treatment, for logistical reasons described above around the critical need for *ex vivo* screening of clinical samples with short notice requiring the use of pre-printed drug plates, we therefore proceeded to adopt condition 1 for future GliExP development.

To further confirm that our *ex vivo* screening platform was able to give robust and accurate readouts of TMZ sensitivity, we tested its ability to predict MGMT promoter methylation status; a well-established clinical feature of around 40-50% of all glioblastomas that epigenetically silences the expression of the key O6-methylguanine detoxifying enzyme MGMT. ^37^

**Figure 6.**
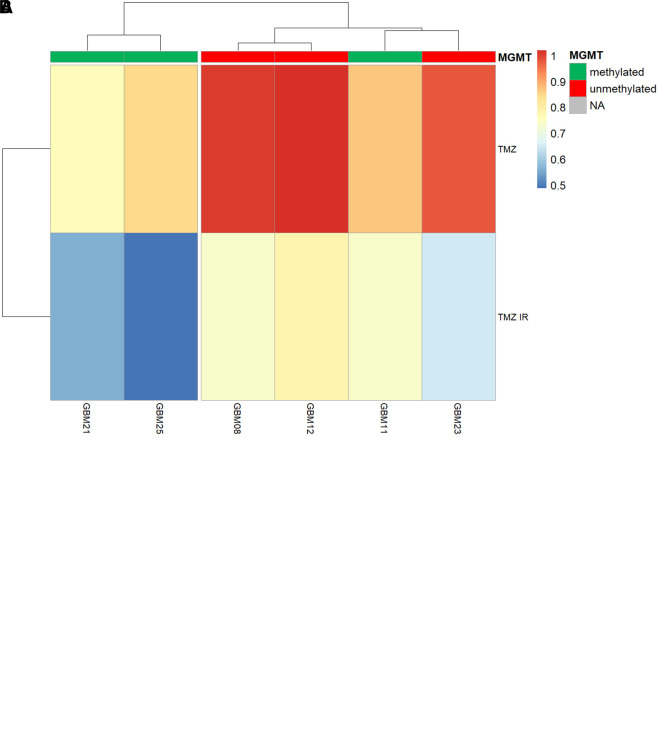
Discrimination of MGMT promoter methylation status within primary GSCs using temozolomide sensitivity within GliExP. A: Comparing the temozolomide (TMZ) LD _50_ values of OX5 (MGMT+/unmethylated) and CX18 (MGMT-/methylated) GSCs with differential MGMT methylation as defined by clinical methylation sensitive pyrosequencing of the MGMT gene promoter in parental tumour tissue. MGMT methylated CX18 GSCs exhibited a significant increased sensitivity to TMZ. Data shown represent median and the 25th and 75th percent quartiles (box) median (middle line). The lower and upper whiskers show the smallest and largest values within 1.5x of the interquartile range below the respective 25th and 75th percentiles. Median LD _50_ are 46.13 ± 60.5 μM (standard deviation) compared to MGMT unmethylated GSCs; LD _50_ = 385.72 ± 102.5 μM (standard deviation). Data shown is derived from three independent biological repeats from two different patient derived GSC models from separate passages (each containing four technical replicates); CX18 and OX5. B: Heatmap showing AUC responses to TMZ alone or in combination with 2Gy IR for 6 different tumours assessed by GliExP. Also shown is their MGMT methylation status which was subsequently determined clinically. GliExP was able to accurately predict the MGMT status for all these tumours based solely on TMZ sensitivity prior to MGMT status being known.

TMZ sensitivity was therefore assessed in an MGMT+ (unmethylated) GSC (Ox5) and a clinically determined MGMT methylated GSC (CX18). Note that CX18 has been deem as “equivocal” methylation status based on 10.7% exhibiting methylation across all CpG islands analysed (clinical data not shown), and therefore could be considered on the weaker side of MGMT methylation and subsequent TMZ sensitivity. However, GliExP was able to distinguish significant TMZ sensitivity between OX5 and CX18 GSC which correlated with their MGMT methylation status ( Figure 6A). Importantly, when analysis of further primary GSCs was carried out before clinical MGMT status was known, the resulting TMZ sensitivity allowed us to predict with 100% accuracy the MGMT status ( Figure 2B).

**Figure 7.**
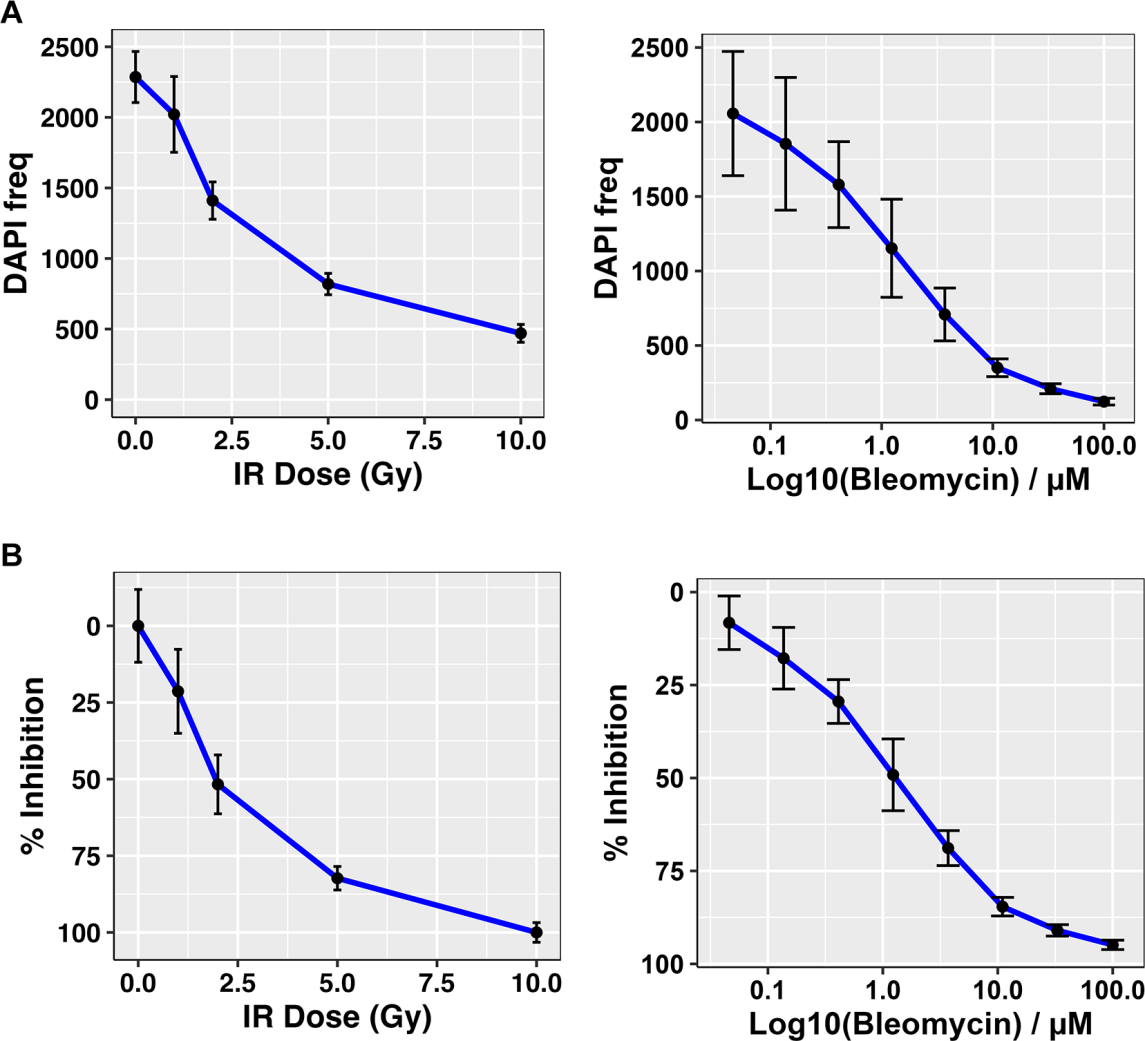
Dose response curves for CX18 GSCs treated with either IR or Bleomycin. A: Cell frequency measured using DAPI positive cells. B: Calculated percentage growth inhibition derived from the same data. The response readout (DAPI count) was collected using automated IF microscopy, the counts were normalized using positive and negative controls on each dose plate to provide the response measure (relative inhibition %). Bleomycin LD _50_ = 6.85 μM which is equivalent to LD _50_ IR – 2Gy. Data shown is the mean derived from 3 independent biological repeats with respective SDs.

In addition to TMZ, the current post-surgical SoC regimen for glioblastomas involves a course of radiotherapy (typically 60Gy over six weeks in 2Gy fractions). ^16^ We are fortunate, and in the minority of research laboratories, in that we have access to an experimental ionising radiation (IR) source within our research facility (~2Gy/min ^137^Cs irradiator; CIS Bio International IBL437c). However, many places that may wish to adopt an *ex vivo* drug screening platform as an alternative to animal-based preclinical models may not have such facilities. We therefore assessed the potential use of the radiomimetic agent bleomycin within GliExP as this can simply be added in a similar manner to small molecule compounds by comparing a dose range against known ionising radiation doses within our experimental Cs ^137^ facility ( Figure 7). For future use and for the benefit of groups that do not have access to a radiation source, we calculated that a concentration of around 6.85μM Bleomycin was equivalent to a 2Gy dose of IR ( Figure 7).

**Figure 8.**
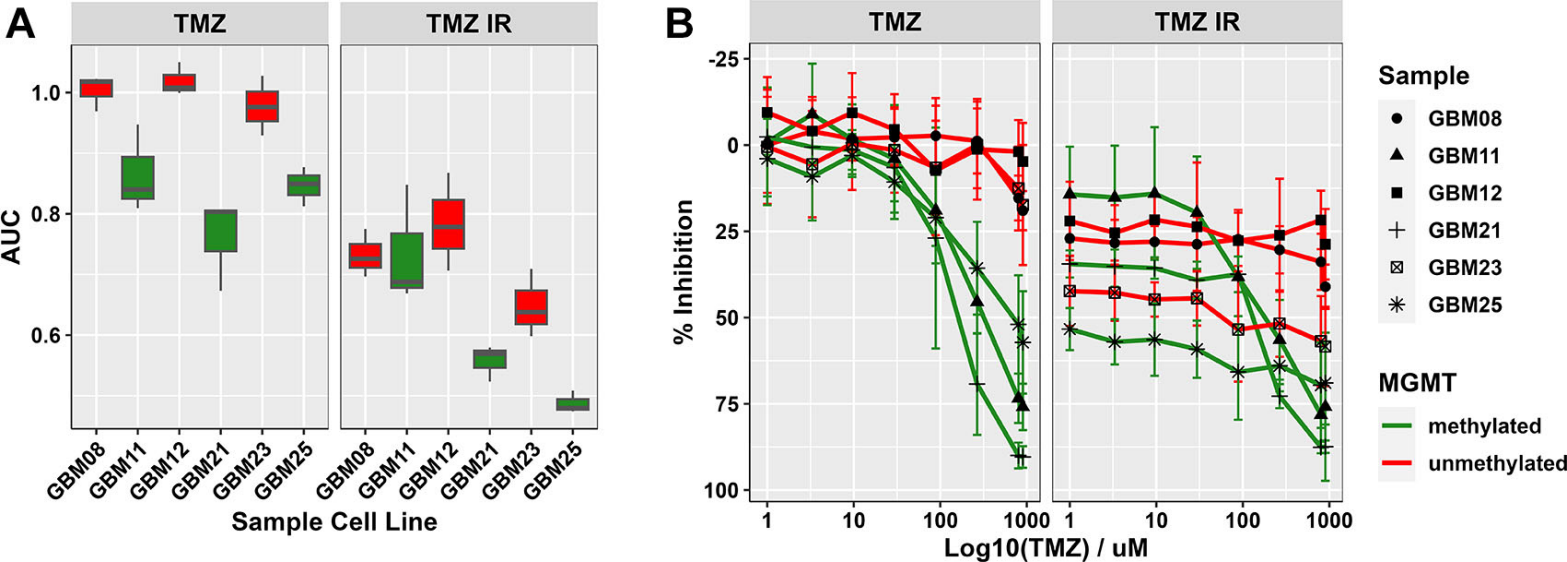
Assessment of combination IR with TMZ treatments in GliExP. A: Box plot comparing the median and interquartile AUC values calculated by GliExP for the indicated GSCs treated with TMZ ± 2Gy IR. Samples shown in green represent methylated MGMT status while red represents unmethylated. B: Line graphs for the same data showing % growth inhibition. All data shown is derived from 3 independent biological repeats (with 4 technical replicates from the same patient derived cell line) with their respective SDs.

As the current SoC for glioblastomas involves a course of radiotherapy alongside TMZ for patients deemed well enough to receive this, we combined IR with TMZ within GliExP. Encouragingly, we were able to determine the combined cytotoxic effects of IR on top of concomitant TMZ across all primary GSC tested to date ( Figure 8).

### Proof-of-concept therapeutic and preclinical compound screening using GliExP

Given the encouraging development and optimisation data around GliExP (see Figures 4- 8), we proceeded to carry out an initial proof-of-concept drug screening. This consisted of creating an initial pre-printed drug plate consisting of just over 30 therapeutic and pre-clinical compounds. The dose ranges for the initial batch of pre-printed GliExP plates we based on existing literature around LD _50_ values across a range of cell lineages, including data from our own laboratories and consisted of four concentrations + a vehicle control for each compound. Using this batch of plates, we proceeded to screen GSCs derived from 18 individual patients taken straight from surgery, including a couple of multi-region samples to assess potential differential drug response associated with spatial heterogeneity, a known driver of therapeutic resistance in the clinic ( Figure 5).

**Figure 9.**
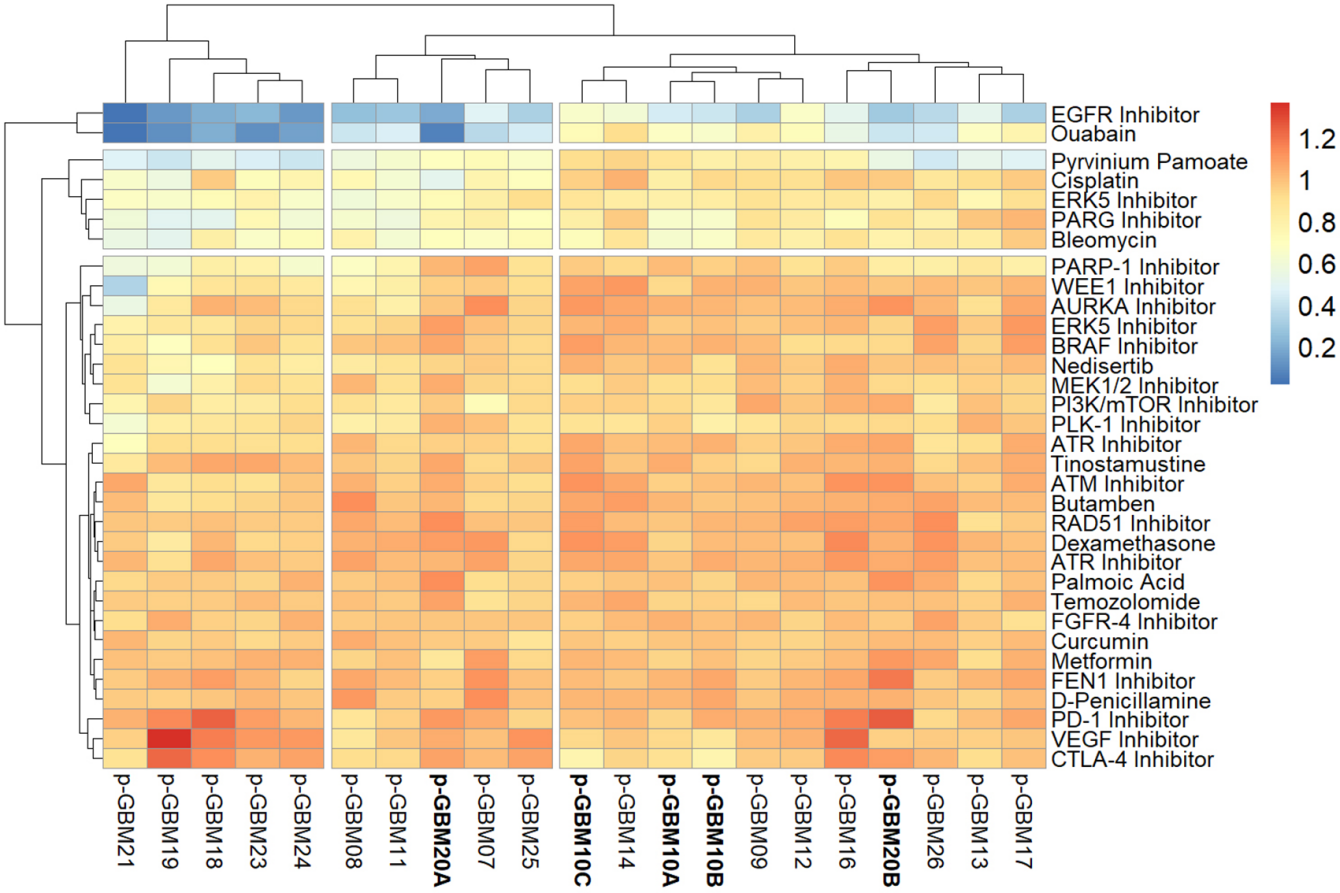
GliExP heatmap depicting raw AUC drug response for primary GBMs dissociated from 18 individual tumours within a week of surgery. Scale shown represents cell death as 0 (blue) and cell survival as 1 (red). Larger samples such as GBM10 (A, B and C) and GBM20 (A and B; indicated in bold) were sectioned into multiple regions and screened independently in order to highlight the intertumoral heterogeneity of the disease. AUC values were calculated from 2 technical replicates following 96hr drug incubations using R package GR metrics and the heatmap was constructed using R package pheatmap as detailed in the methods section. Note, due to the fact that this was performed on limited fresh clinical material, biological repeats were not performed.

This initial set of encouraging data demonstrates that GliExP is able identify potential differential drug sensitivity for a given individual tumour using freshly derived GSC populations from surgically resected tumour within a week of surgery taking place. The limited number of multi-region samples on this initial screening plate also suggests that GliExP is able to determine differential drug sensitivity within a given tumour, further highlighting how spatial heterogeneity can lead to therapeutic resistance. It is important to note that the perceived universal inhibitory effects of the EGFR inhibitor are likely an artifact of the EGF-conditioned media used to enrich/grow GSCs. Encouragingly, we observe that some GSCs are sensitive to a number of compounds with related targets, such as GBM21 being sensitive to both WEE1 and Aurora kinase inhibitors, and GBM19 being sensitive to both PARP1 and PARG inhibitors ( Figure 9). However, moving forward, longer incubation times and extended concentration ranges would be beneficial for certain compounds to allow a deeper assessment of inhibitory effects and calculation of more accurate LD _50_ values (see Discussion section for further details).

**Figure 10.**
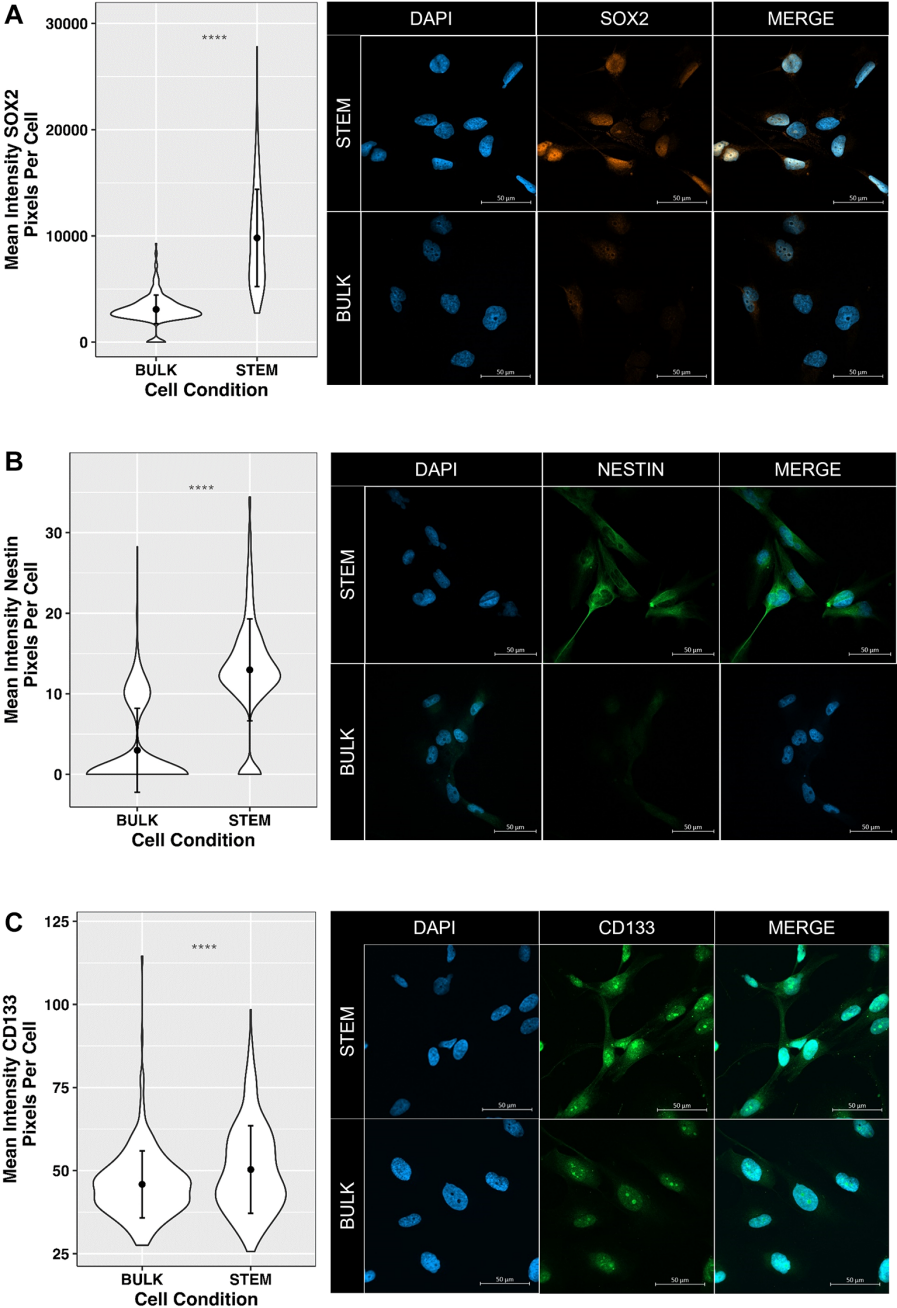
Initial optimisation of GSC marker expression within GliExP to determine potential GSC-specific drug efficacy. A: Left panel; violin plots showing mean fluorescence intensity of Sox2 expression (pixels/cell) in both bulk and stem enriched CX18 GSCs. Right panel; example images. B: and C: Same as in A but showing data for Nestin and CD133 respectively. Data shown are means derived from three independent biological repeat experiments from separate passages of matched ‘bulk’ and ‘stem’ derived CX18 cells with their respective SDs. Each biological repeat experient contained analysis of 100 individual cells. Statistical analysis was performed using Man-Whitney U test to highlight significant differences GSC marker staining intensity between bulk and stem populations (p < 0.001).

Finally, in order to further develop GliExP to be able to identify potential GSC-specific compound cytotoxicity within the cell milieu, we have carried out initial staining optimisation of the GSC markers Sox2, Nestin and CD133 using direct fluorescent conjugated antibodies. To determine GSC specificity of the staining, we cultured CX18 cells as either tumour bulk or GSC-enriched populations (see methods section) prior to staining ( Figure 10). Across multiple optimisation experiments, we found that the most robust of these GSC markers within GliExP were Sox2 and Nestin ( Figure 10A and 10B), and that these correlate with mRNA expression levels ( Figure 2). It is interesting that in our hands, CD133 was not as robust a GSC marker as one would have predicted based on mRNA expression data ( Figure 2). However, there is evidence within previous studies that CD133-negative cells can be labelled incorrectly due to specific antibodies only targeting the AC133 specific epitope, which is located in one of the extracellular domains of membrane-bound CD133 (Barrantes-Freer *et al.*, 2015). We therefore would need to test additional CD133 antibodies within our platform to ascertain if this is indeed the case. However, the robust ability of both Sox2 and Nestin to distinguish GSC-enriched populations gives us confidence that these could be implemented in future iterations of GliExP.

## Discussion

The devastating prognosis of patients with glioblastoma has not improved significantly over the last 50 years despite the use of cell lines, more complex co-culture/microenvironment systems, and a range of animal-based models. ^3^ This highlights a need to explore novel strategies such as *ex vivo* drug screening to try and improve treatment outcomes for these patients. ^19^ Considering the extreme intertumoral heterogeneity of the disease, it seems impossible to expect a one-size-fits-all treatment regime to be successful to treat all glioblastomas. As such, *ex vivo* drug screening aims to discover successful individualised niche treatments for patients and embed assessment of intra- and intertumoural heterogeneity early within preclinical drug evaluation. Such compounds can include well-established/safe therapeutics used to treat other cancers and diseases, but *ex vivo* platforms also facilitate the rapid assessment of novel preclinical agents as a potentially superior replacement (speed, affordability, tissue-sparing, human-specific and more humane) for traditional preclinical animal models.

Current clinical regimens treat all glioblastoma patients with TMZ and/or IR irrespective of MGMT status, even though around half of glioblastomas are unmethylated and may not significantly benefit from TMZ when overall survival rates are considered. ^38^ Indeed, within clinical trials it has been shown acceptable to remove TMZ from the treatment regimes in unmethylated MGMT patients, as long as the patient is offered an alternative treatment method. ^39^ *Ex vivo* drug screening has the potential to be adopted for such patients to help guide this ‘alternative treatment’ regime, discovering patient/tumour specific drug sensitivities. As shown here, GliExP has the potential to rapidly identify TMZ responsive/non-responsive tumours ( Figure 6) and can even be used to identify potential alternative treatment strategies alongside existing SoC therapies such as radiotherapy ( Figure 8), that would often be a pre-requisite of a clinical trial in the setting of newly diagnosed glioblastoma.

A major benefit of *ex vivo* drug screening is its speed, where results can be obtained within a week of surgery, which is within the clinically relevant window to a patient’s first line of treatment following surgery. Potential drug hits can be then evaluated by a multi-disciplinary team (MDT)/clinical decision board (consisting of oncologists, pharmacists, pathologists, and clinical scientists) alongside discussion of the formal histopathological diagnosis and subsequently prescribed depending on the safety and patients health condition, similar to previous work achieved in haematological cancer. ^40^ Due to the classification as moderate-severe under the Animals in Scientific Procedures Act, it could be deemed unethical for PDX models to be generated on such a large scale when any potential drug hits could not subsequently be confirmed clinically, considering the average life expectancy of glioblastoma (12-18 months) and turnaround time for successful PDX engraftment of between four to eight months. ^37^^,^^41^ Advances into Mini-PDX models, which are generated through injection of patient tumour cells into immunocompromised mice within special hollow capsules in order to increase turnaround and reduce complexity, could however circumvent these issues and deliver such data in a much sorted time frame than traditional PDX models (~one week). ^42^^–^^44^ However, the ethical issues around the volume of animals needed in order to assess a large range of therapeutics alone or indeed in combination still remain. *Ex vivo* screening platforms such as GliExP could therefore facilitate new individualised treatment regimens to be implemented straight away, alongside and or replacing current standard of care therapy.

Although extremely promising, and with some exciting proof-of-concept studies recently completed, we are still in the early stages of *ex vivo* screening platform development, certainly for solid tumours. ^19^ To our knowledge, the work presented here represents the first such attempts to develop a robust *ex vivo* drug screening platform for gliomas using freshly dissociated tumour tissue. We believe that we have optimised the seeding order/conditions, growth, imaging and analysis to allow us to screen almost all glioma samples received from the clinic either against bespoke drug plates or current SoC treatments ( Figures 8 and 9). We believe that we are now at a stage where we can also start to combine drug plate screening with SoC therapies such as IR (untreated plate *versus* 2Gy treated plate) to mimic how these tumours are treated in the clinic and within clinical trials (certainly if TMZ can be removed from the treatment strategy for identified MGMT unmethylated tumours; see above). As we move forward with further iterations of GliExP, we are hoping to further refine multi-channel imaging to allow us to identify compounds/treatments that exhibit specific potency against the GSC population whilst sparing other cell types ( Figure 10). In tandem with this, we are also working with computer scientists and artificial intelligence experts to try and develop machine learning AI technologies that can further automate and enhance speed/efficiency at which GliExP can operate. Additionally, we are working within the larger *Ex vivo* determined cancer therapy (EVIDENT; NCT05231655) team with Sheffield (based at the University and Hospital sites) to knowledge share our *ex vivo* experiences to help develop additional animal-sparing *ex vivo* screening platforms for other solids tumours ( *e.g.* bladder, kidney, head and neck etc).

From a 3Rs perspective *ex vivo* has the potential to become a preclinical alternative, replacing and reducing the requirement for murine avatars for testing experimental therapeutics, not only in glioblastoma but for many other solid malignancies. Our first-generation drug plates contain 35 different therapeutics which have been used to screen 18 different patient samples ( Figure 9). Feasibly it would be impossible to create the numbers of avatars needed per patient to obtain all 35 different drug response profiles using traditional PDX models. On average 5-10 mice are required to test one single treatment, to recreate this work *in vivo* a minimum of 3,150 PDX models would be required (175 models per patient). This is an especially large sample, and the time and cost of serially transplanting into multiple cohorts of mice would be astronomical, and extremely time consuming. Additionally genetic drift of the tumours through multiple cohort passages has resulted in engraftment being restricted to 10 or fewer passages, ^45^ therefore limiting the drug screening capability of PDX models.

Based on a comprehensive analysis of current global *ex vivo* studies utilising murine avatars (see Table 2 of our recent review article ^19^), together with data obtained from the GlobalData database around the current therapeutic development for glioma, we estimate that around 400+ mice are used per study, and ~20,000+ mice were used globally in this setting over the last 5 years. We therefore believe the application of GliExP could replace the use of ~2,000 mice per year, with the potential for expansion to other solid tumour types. This estimate increases significantly when one considers our plans to combine multiple therapeutics in tandem with current SoC radiation and TMZ treatments. To facilitate this, we are currently in discussions with our research ethics and Healthcare Gateway teams at the University and Royal Hallamshire Hospital to explore how we could develop GliExP into a research, clinical and commercial service given recent positive conversations with drug development companies.

## Data Availability

Figshare: Ex-vivo drug screening of surgically resected glioma stem cells to replace murine avatars and provide personalise cancer therapy for glioblastoma patients, https://doi.org/10.15131/shef.data.c.6710475.v1. ^46^ This collection contains the following underlying data projects:
- IF Mean Intensity files for markers CD133, Nestin, SOX2 and Vimentin (Datasets) - CX18 Bulk vs Stem qPCR Analysis - CX18 Bulk Vs Stem CD133 IF image files - CX18 Bulk Vs Stem Nestin IF Image Files - CX18 Bulk Vs Stem SOX2 IF Image Files - CX18 Bulk Vs Stem Vimentin IF Image Files - Western blots for CX18 undifferentiated (primary stem), differentiated (bulk) and de-differentiated (secondary stem) Data are available under the terms of the Creative Commons Attribution 4.0 International license (CC-BY 4.0).
