# Supplementary material for: Ex-vivo drug screening of surgically resected glioma stem cells to replace murine avatars and provide personalise cancer therapy for glioblastoma patients
Source: F1000Res. 2024 Mar 11;12:954. Originally published 2023 Aug 8. [Version 2] doi: 10.12688/f1000research.135809.2 (PMC10548111; doi:10.12688/f1000research.135809.2)
Supplement: Peer review history

# Reviewer report: Reviewer response for version 2

**DOI:** 10.5256/f1000research.163477.r254519

**Published:** 2024-03-18

**Authors:** Natividad Gomez-Roman

**Affiliations:** 1 Department of Pharmacy and Biomedical Sciences, University of Strathclyde, Glasgow, Scotland, UK

No further comments.

Is the rationale for developing the new method (or application) clearly explained?

Yes

Is the description of the method technically sound?

Yes

Are the conclusions about the method and its performance adequately supported by the findings presented in the article?

Partly

If any results are presented, are all the source data underlying the results available to ensure full reproducibility?

Yes

Are sufficient details provided to allow replication of the method development and its use by others?

Yes

Reviewer Expertise:

Glioblastoma, preclinical in vitro and in vivo models of cancer, high-throughput assays

I confirm that I have read this submission and believe that I have an appropriate level of expertise to confirm that it is of an acceptable scientific standard.

# Reviewer report: Reviewer response for version 1

**DOI:** 10.5256/f1000research.148941.r195890

**Published:** 2023-10-03

**Authors:** Anke Brüning-Richardson

**Affiliations:** 1 School of Applied Sciences, University of Huddersfield, Huddersfield, England, UK

The article by Gagg *et al.* clearly describes a novel *ex-vivo* drug screening platform for personalised cancer therapy for glioblastoma patients. The authors are making a strong case to reduce animal-based pre-clinical models and replace mouse orthotopic/cranial resection models to ascertain drug responses in patient-derived glioma stem cell populations. 

 There are a few points to consider for further discussion.

1. Can the authors further comment on the problems associated with the use of PDX models in terms of different methodologies and protocols used by researchers in the field? Unified protocols and sharing of the same resources (i.e. patient-derived cell lines) would  generate more meaningful data, but this is currently not common practice. 
2. Apart from the described novel *ex vivo* drug screening platform I think it would be worth mentioning that there are also additional, advanced 3D in vitro models and microfluidic Lab-on-a-chip technologies which may complement the research described here, which will help reduce the number of animals used in glioblastoma research.
3. Can the authors comment on published data from *in vivo *models and the outcomes of research into some of the drugs they were investigating here, if available? How do the two models compare?

Just some minor typos or inconsistencies in the text:

- Page 11: 2 'pre warmed' should be 'pre-warmed'.
- Page 11: 2.. Full stop after 'PBS'.
- Page 13. 20. Full stop after 'tissue'.
- On page 13, follow on instructions, for consistency there should be full stops after each sentence.
- Also at the end of the note, so after 'GSC'.
- Page 15: 2 Full stop after 'stage'.
- Page 18 - Results section - 'condition 1' should be 'Condition 1', to keep it consistent, so capital letters for this.
- Page 19. Figure 5 figure legend: 'condition 3' should be 'Condition 3'.
- Figure 6 figure legend - Temozolomide should be TMZ as already mentioned previously.

Is the rationale for developing the new method (or application) clearly explained?

Yes

Is the description of the method technically sound?

Yes

Are the conclusions about the method and its performance adequately supported by the findings presented in the article?

Yes

If any results are presented, are all the source data underlying the results available to ensure full reproducibility?

Yes

Are sufficient details provided to allow replication of the method development and its use by others?

Yes

Reviewer Expertise:

Glioblastoma, pre-clinical models, drug development

I confirm that I have read this submission and believe that I have an appropriate level of expertise to confirm that it is of an acceptable scientific standard.

# Reviewer report: Reviewer response for version 1

**DOI:** 10.5256/f1000research.148941.r195888

**Published:** 2023-08-31

**Authors:** Natividad Gomez-Roman

**Affiliations:** 1 Department of Pharmacy and Biomedical Sciences, University of Strathclyde, Glasgow, Scotland, UK

A meticulously crafted manuscript delineates a high-throughput screening platform designed for the assessment of novel treatment combinations employing ex vivo patient-derived glioma tumours. The document is well-written, furnishing a comprehensive exposition of the methodology.

 Some minor aspects necessitate revision:

1. Regarding all drug dose-response curves: a) It is imperative to incorporate the control value (no drug) in these graphs. As 'X' employs a logarithmic scale, this can be indicated by a disjointed line, creating a visual gap between the 0 drug value and the first lowest dose. b) The provision of confidence intervals is indispensable for all LD50 data. c) The exhibited curves are not intrinsically fitted to the dose-response model. Although I acknowledge that the AUC is derived from these graphs, for precise LD50 calculations accompanied by confidence intervals, I propose appropriately fitting the data for both presentation and LD50 calculation. My recommendation is to employ a 4-parameter dose-response curve, utilizing the 'drc' package in R (version 3.6.3), available at ( https://www.R-project.org).
2. In Fig. 8 – Could you specify the concentration of TMZ utilized for the box plots in section A?
3. Given the intricate cellular composition of freshly excised tumour biopsies, encompassing tumour cells along with associated elements like microglia, it is imperative to acknowledge that the cellular populations within GliExP would manifest heterogeneity. This factor could potentially introduce confounding variables to the outcomes of the screening, as responses may emanate not solely from glioblastoma cells, but also from the surrounding glioblastoma-associated cells. Could the authors kindly furnish insights into the viability of other cell types, such as microglia, within the GliExP models? If these cell types are indeed present, it would be valuable if the authors could provide an estimate of the percentage representation of each cell type cultivated in the GliExP model.
4. With reference to the CSC markers, could the authors elucidate the proportion of cells retaining these markers, and subsequently, establish a correlation with the characteristics of the biopsied material? Is there a discernible correlation between these values?
5. Rectification is required in the legend of Fig. 10 – a typographical error is apparent in the penultimate sentence, where 'experiment' is misspelt. Kindly effect the necessary correction.

Is the rationale for developing the new method (or application) clearly explained?

Yes

Is the description of the method technically sound?

Yes

Are the conclusions about the method and its performance adequately supported by the findings presented in the article?

Partly

If any results are presented, are all the source data underlying the results available to ensure full reproducibility?

Yes

Are sufficient details provided to allow replication of the method development and its use by others?

Yes

Reviewer Expertise:

Glioblastoma, preclinical in vitro and in vivo models of cancer, high-throughput assays

I confirm that I have read this submission and believe that I have an appropriate level of expertise to confirm that it is of an acceptable scientific standard.
